# Investigation on the Mechanical Properties of SMA/GF/Epoxy Hybrid Composite Laminates: Flexural, Impact, and Interfacial Shear Performance

**DOI:** 10.3390/ma11020246

**Published:** 2018-02-06

**Authors:** Shicheng Zhao, Jianxin Teng, Zhenqing Wang, Xiaoyu Sun, Bin Yang

**Affiliations:** 1 College of Aerospace and Civil Engineering, Harbin Engineering University, Harbin 150001, China; 13654574602@163.com (S.Z.); tengjianxin123@163.com (J.T.); wangzhenqing@hrbeu.edu.cn (Z.W.); 2 School of Mechanical and Power Engineering, East China University of Science and Technology, Shanghai 200237, China

**Keywords:** SMA wire, flexural test, low-velocity impact test, interface property

## Abstract

In this article, hybrid composite laminates with shape memory alloy (SMA) and glass fiber (GF) as the reinforced phase, and epoxy resin as the host material, were manufactured by vacuum-assisted resin injection (VARI) processing. The SMA wires were embedded into the GF/epoxy composites with three kinds of modes. The effect of SMA content and the position on the flexural, low-velocity impact performance of the hybrid composite laminates was investigated. It was found that the bonding performance between the SMA wire and the host material is the key factor that determined the final overall performance of the hybrid composite laminates in both the static and dynamics tests. Based on these experimental phenomenon, we further carried out the fiber pull-out experiment to improve the interfacial shear strength between the SMA and epoxy resin. It was found that the interfacial performance could be enhanced significantly by adding nanoparticles in the interface phase.

## 1. Introduction

Laminated composite plates show increasing application in industry due to their high stiffness and strength-to-weight ratio. Application of shape memory alloys (SMA) has increasingly attracted attention in smart structures, compared with other advanced smart materials, due to their shape memory effect (SME) and superelasticity (SE) that are associated with the reversible phase transformations between austenite and martensite [1]. Due to the ability of recovering extremely large strains, SMA is usually used in engineering fields, especially in the aerospace industries [2]. Embedding SMA actuators into polymer matrix composites could form SMA composites. This material is a new type of smart hybrid composite structure. In SMA composites, SMA can be deformed and returned to its original shape by its phase transformation. As common fiber-reinforced plastic composites (FRPs), SMA composites are highly susceptible to internal damage, which is generally caused by external static and dynamic mechanical behaviors, such as the flexing, fatigue, and the low-velocity impact on composite structures [3,4,5,6,7,8,9]. All the load could cause barely-visible damages in SMA composites, and the surface may appear to be safe and sound upon visual inspection. Therefore, it is valuable to investigate the static and dynamic mechanical performances of SMA-based hybrid composites to expand their application scopes.

Many studies regarding dynamic and static responses of SMA-based hybrid composite structures have been reported, in which the thermo-mechanical characteristics of SMAs are extracted from the approximate data. For instance, Zhang et al. [10] fabricated epoxy resin composites with Ni-Ti alloy short fibers, and they also investigated their mechanical properties. The results showed that the flexural rigidity of SMA/epoxy composites increases owing to the addition of SMA fillers. Samadpour et al. [11] investigated the nonlinear free vibration of thermally-buckled sandwich plate with embedded pre-strained shape memory alloy (SMA) fibers in temperature-dependent laminated composite face sheets. The one-dimensional Brinson model is employed for recovery stress generated during phase transformation of the pre-strained SMA fibers. Nonlinear equations of motion of sandwich plate are derived based on the first-order shear deformation theory and von Karman geometric nonlinearity via the Hamilton principle. As the substructure of a vibration energy harvester, Lu et al. [12] designed shape memory alloy (SMA)-reinforced epoxy composites. By measuring the vibration mode of the energy harvester, they discussed the influence of SMA-epoxy and temperature on the dynamic response. Kabir et al. [13] presented an analytical study on thermal, mechanical, and thermomechanical buckling and post-buckling of symmetric laminated composite plates reinforced with shape memory alloy (SMA) fibers. Li et al. [14] proposed a novel variable stiffness finger actuated by heating the embedded SMA fibers. Zheng et al. [15] described a numerical framework to simulate the fatigue crack growth of single edge-notched steel elements patched with shape memory alloy (SMA)/fiber reinforced polymer (FRP) composite patches. The small crack propagation stage was simulated using the analytical model based on the equivalent initial flaw size method. In terms of the micro-mechanical viscoelastic properties of SMA/GF/epoxy hybrid composite materials, Rana et al. [16,17] discussed the differences in mechanical behavior between alternating and random styrene-methyl methacrylate copolymers. Tian et al. [18] comparatively investigated the fracture toughness of Ti-Al metal-intermetallic-laminate (MIL) composite with and without Ni-Ti shape memory alloy fibers via a three-point bending test. Their results indicated that the fracture toughness of the Ni-Ti shape memory alloy fiber-reinforced (SMAFR) Ti-Al MIL composite was almost twice as much as that of the Ti-Al MIL composite without Ni-Ti fibers.

Another aspect, even though SMA wire reinforced polymer-based composites have been recognized as an excellent type of smart materials, is that the interfacial bonding strength between the wire and its surrounding matrix is weak, which limits its applications in many engineering industries. The overall mechanical properties of SMA composites also depend on fiber, matrix, and fiber/matrix interface. Compared with individual fiber and matrix mechanical properties, interfacial load transfer performance plays a more important role. Generally, factors including local stress, matrix residual stress, and the applying of an external load mode could affect the interfacial load transfer performances [19]. Weak interfacial bonding will lead to lower shear stress transfer efficiency and, consequently, unfavorably influences the overall composites strength. Therefore, improving adhesion between fiber and matrix is essential to obtain optimum SMA composite properties. Yuan et al. [20] introduced a mechanical indentation method to effectively enhance the interfacial bonding strength of SMA composites. According to their results from a wire pullout test, the interfacial bonding strength of an indented SMA wire composite increased by 4.48–8.58 times as compared with a hand-sanded SMA wire composite under room temperature conditions. Choi et al. [21] assessed the pullout resistance of superelastic shape memory alloy (SMA) short fibers with different end shapes, which provide different anchoring actions, through monotonic and hysteretic pullout tests. Their results showed that the maximum pullout resistance of the spearhead fiber was 3.74 times that of the L-shaped fiber.

Despite of the advanced in enhancing the macroscopical and interfacial mechanical performance of SMA reinforced composites, the mechanical performance of SMA composites still needs further investigation to extend their application scopes in industry fields. The objective of this paper is to study the static and dynamic mechanical properties of SMA composites. The hybrid composite laminates with shape memory alloy (SMA) and glass fiber (GF) as reinforced phase and epoxy resin as the host materials were manufactured using vacuum-assisted resin injection (VARI) processing. The SMA wires were embedded into the GF/epoxy composites with three kinds of modes. The effects of SMA wires content and position on the flexural, low-velocity impact performance of the hybrid composite laminates were investigated. Fiber pull-out experiments with focus on improving the interfacial shear strength between SMA and the epoxy resin were further carried out.

## 2. Manufacturing and Test Methods

### 2.1. Materials and Manufacturing

**Figure 1:**
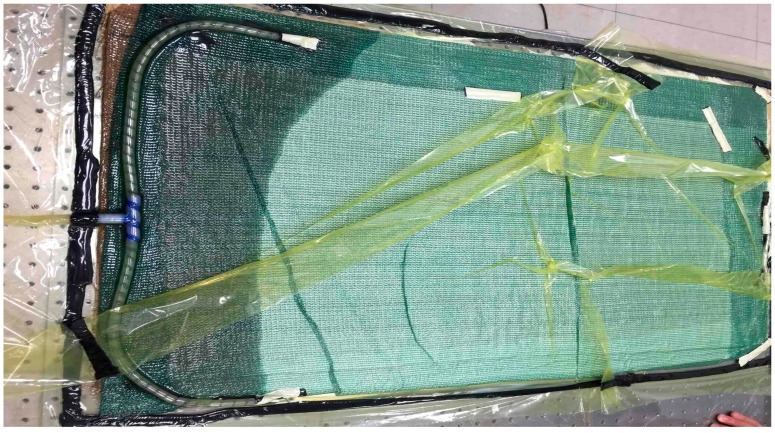
VARI processing adopted to manufacture SMA/GF/epoxy hybrid composite laminates.

**Figure 2:**
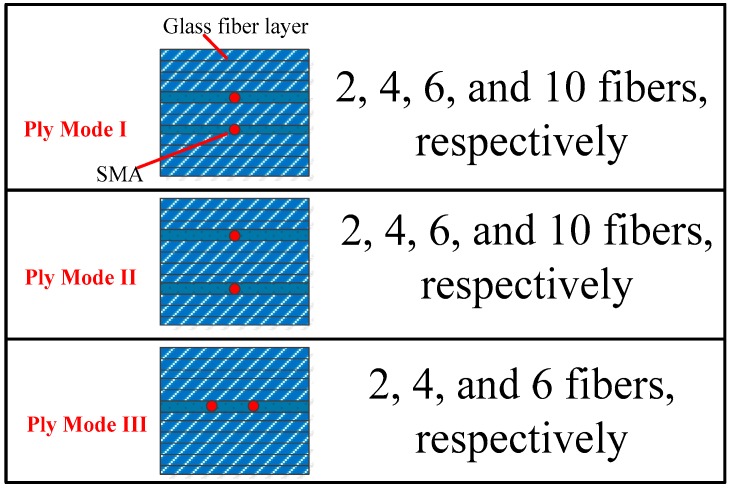
SMA location and their numbers in the composite laminates.

The composite laminates were manufactured by vacuum-assisted resin injection (VARI) processing. Figure 1 indicates the schematic diagram of the VARI setup. It should be noted that the final dimension of the obtained SMA/GF/epoxy hybrid laminates is 800 × 400 × 2.4 mm with a ply style of [90°/0°]_10_. During the processing, a glass square plate was placed at the bottom as holder, and then release film was bonded on the surface of the glass. The composite laminate was placed on the release film, and then covered with peel ply and silk ply. A total of 11 kinds of laminates were prepared in the experiment, and the stacking sequence of the unidirectional glass fiber cloths and SMA were in according with the schematic diagram shown in Figure 2. Then the laminate was sealed in a vacuum bag with sealant tape. To ensure that the resin could flow uniformly, a delivery pipe was fixed at the entrance. After resin infusion, curing at ambient temperature and a vacuum level of 600 mbar was performed for 24 h.

### 2.2. Flexural and Low-Velocity Impact Test

To characterize the mechanical behavior of SMA composite laminates, five types of bending specimens were cut from the square laminate by a low-speed diamond saw blade cutting machine. The specimens’ structures can be found in Figure 2. A displacement controlled test with stroke rate 3 mm/min was performed on a universal testing machine characterized by a maximum loading capacity of 100 kN. The dimension of the specimen was 80 × 25 × 2.4 mm according to the ASTM standard. Tests were performed at room temperature (293.5 K ± 1 K), with five specimens per type. Flexural elastic modulus *E_b_* and strength *σ_b_* were calculated from the obtained load-displacement curves using the following formulas:

(1) $$E_{b}=\frac{l^{3}\Delta F}{4bh^{3}\Delta d}$$

(2) $$\sigma_{b}=\frac{3F_{b}l}{2bh^{2}}$$

where *b* is the specimen width, and *h* is the specimen thickness; *l* is the span length, and *F_b_* is the failure load. Δ*F*/Δ*d* is the slope of the initial straight-line portion of the load-deflection (*F*-*d*) curve.

**Figure 3:**
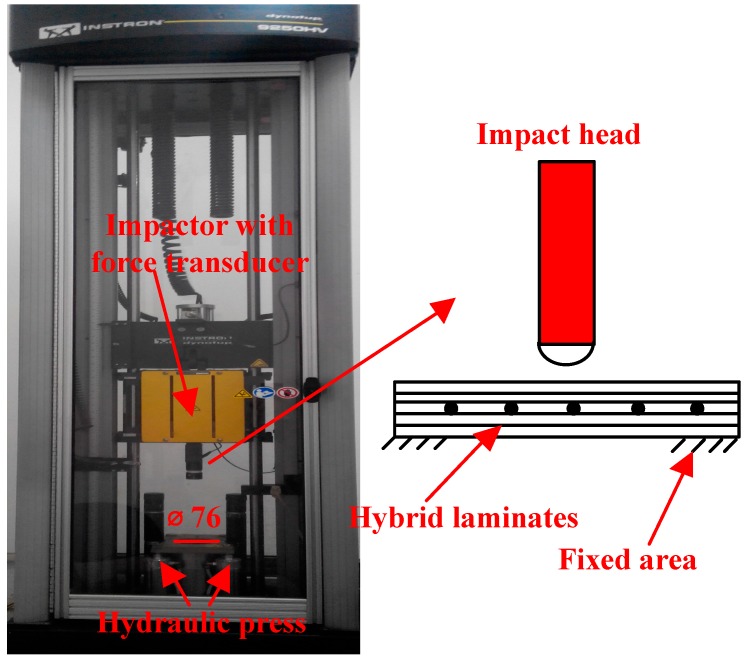
Instron Dynatup 9250HV drop weight impact testing machine.

To study the dynamical properties of SMA laminates, the low-velocity impact test were carried out. The specimens with dimension of 100 × 100 × 3 mm were used. The low-velocity impact test was performed at room temperature by an Instron Dynatup 9250HV drop weight impact testing machine (Instron Corporation, Boston, MA, USA), as shown in Figure 3. The machine consists of a pneumatic clamping fixture, a drop hammer device, and a data acquisition system. The clamp is comprised of two circular rings with a diameter of 76 mm. A steel hemispherical impact head and a few pieces of metal plates are the components of the drop hammer device. Once impact begins, the drop hammer device will drop from a predetermined height and the steel hemispherical impactor will hit the center of the test sample between the round-clamped panels. The drop hammer device is guided through two smooth columns, and it can automatically rebound after its initial contact, avoiding restrikes. The diameter of steel hemispherical impactor is 14 mm. During the impact process, the history of impact force is measured by a load cell located above the impactor. The impact velocity is recorded by a pair of photoelectric-diodes, which is attached to the base of the test machine. The displacement of the impactor is obtained by a laser detector, which is attached to the moving impact frame. For each type of the laminates, four specimens were used in the low-velocity tests.

### 2.3. Interfacial Shear Strength Test

**Figure 4:**
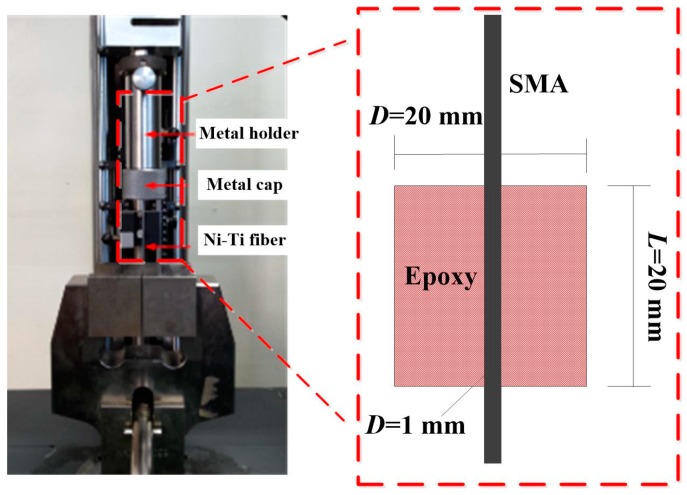
SMA fiber pull-out experiment setup.

The single SMA fiber pull-out test was conducted to investigate the effect of surface adhesion on the pullout strength. The pullout experiment was performed on a Zwick/Roell Z010 (Zwick/Roell, Ulm, Germany) mechanical testing machine at the pullout speed of 1 mm/min. The pullout samples were put in a metal holder to conduct the pullout experiment; the experiment method is shown in Figure 4. One end of the metal holder is a metal cap with a hole 2 mm in diameter in the center, which ensures that the Ni-Ti fiber would not have friction with the cap during the pullout process. The metal holder is attached to the mechanical testing machine through a metal pin. During the experiment, the vinyl ester resin matrix is stuck by the metal cap when Ni-Ti fiber is clipped by a fixture and pulled out from cylinder polymer matrix. The inner diameter of the metal holder is 0.5 mm which is larger than the diameter of the samples, and this guarantees that no radial pressure is produced during the experiment process. Therefore, the pullout test can objectively reflect the results of the adhesive force of interface. All the samples are divided into six groups. Each group contains five samples to ensure that the performance of the specimens can be accurately reflected by the experimental results.

### 2.4. SEM and X-ray Spectroscopy Test

To evaluate the micro-damage morphology of the composites after damage in the mechanical tests, the micro-structure of the fracture region in the composites is observed by scanning electron microscopy (SEM) (Hitachi S-4300, Tokyo, Japan). An X-ray spectroscopy (EDX) (EDAX Inc., Mahwah, NJ, USA) test was conducted to analyze the element distribution in order to investigate the dispersion of nano-silica particles in the interface between SMA wire and the epoxy resin.

## 3. Results and Discussion

### 3.1. Flexural Performance Test

**Figure 5:**
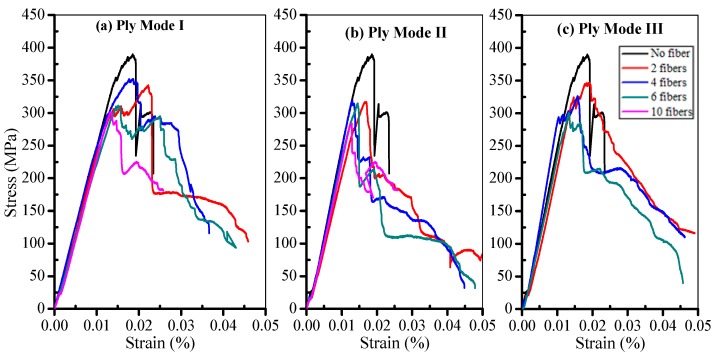
Comparison of the flexural stress–strain curves of SMA composites.

The comparative curves of SMA composites in Ply Mode I to III in Figure 2 are shown in Figure 5. As seen in the Figure 5, the curves firstly increase with strain and then gradually decrease to the minimum value after reaching the peak stress. Table 1 lists the variety of flexural moduli of the composites with different SMA fiber numbers in different ply modes. It is obvious that the flexural modulus of four-SMA-fiber composites has the highest value. The value of flexural modulus of 4-SMA-fiber composites are 23.91, 23.73, and 23.74 GPa, respectively. Compared with GF/epoxy composites, the flexural modulus increase by 5.19%, 4.4%, and 4.44%, respectively. However, six- and 10-SMA-fiber composites present lower flexural modulus than GF/epoxy composites in the table. The increase in the number of fibers enlarge the contact surface of SMA fibers and matrix so that the weak interface between SMA fibers and the matrix has an adverse effect on the properties of the composites which is the discrepancy in material compatibility. However, the flexural strength of the composites are significantly weakened owing to the addition of SMA fibers. The flexural strength of SMA composites is lower than that of GF/epoxy composites. The values of flexural strength are reduced from 391.74 to 368.62 MPa, 369.17, and 318.73 MPa, as shown in Figure 5. This is due to the weak interface between the fiber and matrix, showing a greater effect on flexural strength compared with the effect of SMA. However, a slight increase of the flexural strength is due to the weakening of the effect of the weak interface. The flexural strength of SMA composites were significantly lower than GF/epoxy composites. As mentioned above, the stress concentration exists on the surface of the fiber because of the weak interface. Matrix cracking and delamination in the laminated composites, the debonding between SMA fibers and the matrix, occurred during the flexural performance test. The weak interface can be observed between the SMA fiber and the matrix due to the discrepancy in material compatibility, which further affects the enhancement effect of the composite, especially in the flexural strength aspect of the SMA composites. Moreover, it is concluded that four SMA fibers embedded in the composite is the optimal number, as shown in Table 1 and Figure 5.

### 3.2. Low-Velocity Impact Test

**Figure 6:**
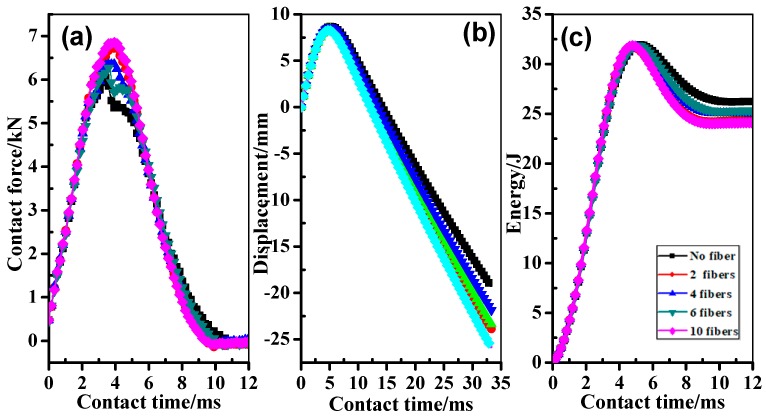
Typical graphs of laminates with one layer of SMAs located at different positions after impact: (**a**,**b**,**c**) are the contact force-time (*F*-*t*) curves, displacement-time (*D*-*t*) curves, and energy-time (*E*-*t*) curves, respectively.

Figure 6 shows the typical curves of laminates with one layer of SMAs inserted in different positions subjected to low-velocity impact with an energy of 28 J. Here, Ply Mode-II was selected as the objective specimens in the impact test. Contact force, as a reaction force of the impactor on the specimen, plays an important role in the analysis of low velocity impact, and the contact force history is obtained by a data acquisition system. The force versus time curves of the laminates are mountain-like shapes, as seen in Figure 6a. It is seen that the tendency is increase sharply and then decrease in the *F*-*t* curves of the laminates. Figure 6b shows the deformation versus time curves of laminates, and the maximum displacement can be evaluated as an essential parameter under low-velocity impact. From *D*-*t* curves in Figure 6b, the laminate with 10 SMAs shows the minimum peak displacement, by contrast, the laminate without SMAs has the maximum peak displacement. In addition, it has been recognized that the deformation energy plays an important role in evaluating the damage process of composite structures under low velocity impact. From the comparison of *E*-*t* curves in Figure 6c, it can be found that the deformation energy of laminate without SMAs has the highest value, while the laminate with 10 SMAs has the lowest deformation energy. According to the data obtained from the low-velocity impact test, it can also be concluded that the laminate can obtain more elastic deformation energy with the assistance of the embedded SMAs.

### 3.3. SMA and GF/Epoxy Bonding Strength Test

**Figure 7:**
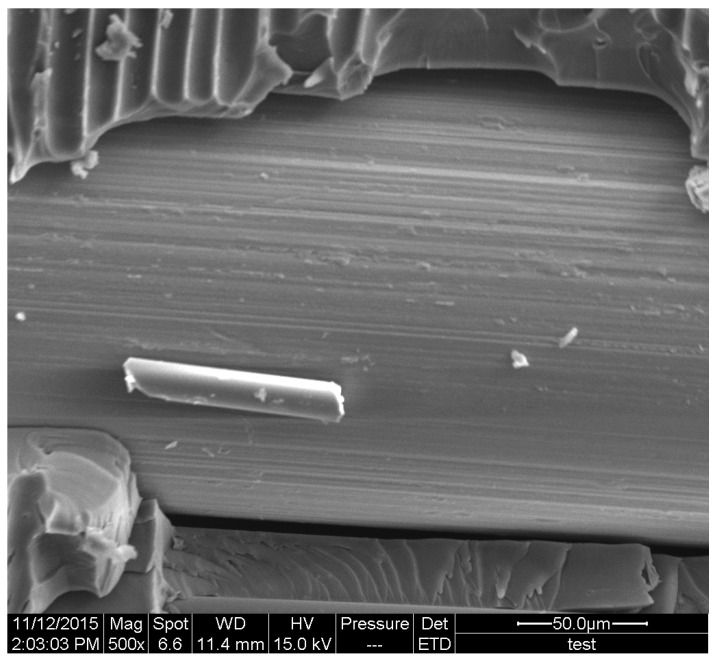
SEM micrographs of SMA in the composites after flexural damage.

**Figure 8:**
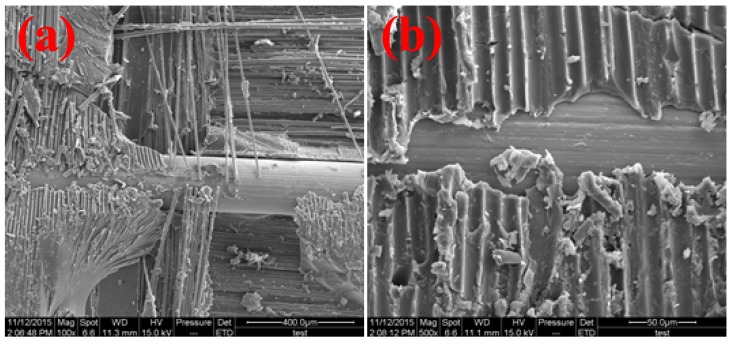
SEM micrographs of SMA in the composites after low-velocity impact damage: (**a**) magnification of 100, and (**b**) magnification of 500.

Figure 7 and Figure 8 illustrate the SEM micrographs of SMA composites after damaged in the flexural and low-velocity impact tests. From the micrographs of the specimens, it can be clearly seen that the bonding performance between SMA and the host materials is very weak. As is known, this weak bonding property would result in lower static and dynamic mechanical performance in the test. Consequently, we are trying to determine an effective approach to enhance the interfacial shear strength of SMA composites in the following work. 

**Figure 9:**
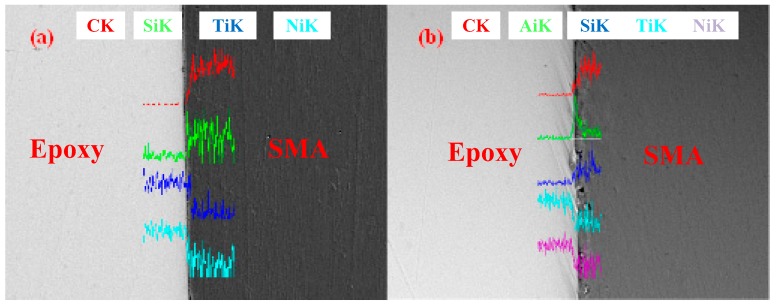
EDX scanning of modified surfaces: (**a**) the initial interface, and (**b**) the nano modified interface.

EDX testing was used to further scan the element distribution in the interface region, shown in Figure 9. Figure 9a shows the element distribution of the interface modified with 2 wt % nano-silica, while Figure 9b shows that of the interface modified with both 2 wt % nano-silica and acid immersion for 8 h. The EDX testing results present that the existence of nano-silica can be observed, which proves that there are nano-particles acting on the interface. In the diagram of the sample modified by both nano-silica nanoparticles and acid immersion, the peak of aluminum is obvious, which is verified by the effectiveness of the adopted PVD method.

Based on the above discussion, the SMA wires were immersed in acid liquid for 0 to 10 h and coated with 2 wt % nano-silica particles. The SMA wire pull-out test results are listed in Table 2. As the wire was pulled gradually in the pull-out test, the load increased with the increase of displacement until complete debonding. After complete debonding, all curves show a sharply-downward trend. Such a characteristic means that the wire was no longer adhered to the host, and it could only bear the external load by friction force at that time. In single-fiber pull-out tests, two situations may appear in terms of the apparent position of the initial microcracks. Microcracks may firstly form in the interface region between fiber and matrix. It is also probable that a microcrack originates in the matrix where stress concentrations are the highest. The cracks then likely reach to the interface and extend along the fiber. In both cases, the crack propagating path can be simply divided into two directions: along the wire length and/or gradually debonding into the radial polymer matrix. Theoretically, Zhandarov et al. [22] have developed a model to describe the current applied force, *F*, as a function of the crack length:

(3) $$F=\frac{\pi d}{\beta }\left\{\tau_{d}\tanh\left[\beta (l_{e}-a)\right]-\tau_{T}\tanh[\beta (l_{e}-a)]\tanh[\frac{\beta (l_{e}-a)}{2}]+\beta a\tau_{f}\right\}$$

where $\tau_{T}$, $\tau_{f}$, and *β* are the residual stress due to thermal shrinkage, the frictional stress in the already-debonded region, and the corrected shear parameter, respectively. *τ_d_* is the shear stress, and *d* is the radius of the matrix; *a* is the radius of fiber, h is the embedded length, and *l_e_* is the calculated radius.

The radial tensile stress, *W_a_*, can be expressed as follow:

(4) $$W_{a}=\sigma_{ult}\lambda$$

where *λ* is the effective normal displacement between the contacting surfaces required for their separation, and *σ_ult_* is the ultimate stress.

As seen in Table 2, SMA wires treated by acid for 8 h and coated with nano-silica particles show the highest interfacial strength. The debonding strength is increased by 52.21%, while the failure displacement is increased by 28.12%. Since micro-nicks were formed by acid treatment and nano-silica was coated on the surface, cured epoxy macromolecules could be protected from tensile breaking during the mechanical tests. Epoxy polymer segments could cover nano-silica particles on the rough fiber surface. These particles reduce the available free volume between the polymer matrix and the SMA surface. Nano-silica particles in the interface region are interlocked with nicks and could hinder macromolecules from moving, and then initial cracks in the material are difficult to form. This micromechanism enhances the critical interfacial shear, which macroscopically appears as the larger pullout load in the test.

## 4. Conclusions

In summary, the flexural and low-velocity mechanical performances of SMA-reinforced GF/epoxy composite laminates are investigated. The effect of SMA content and position on the mechanical performance of the composite are studied. It can be concluded that the addition of SMA wires in GF/epoxy composites has the ability of enhancing the static and dynamic mechanical performance of the obtained composites. The optimal SMA content and position were determined to achieve the hybrid composites with highest mechanical performance. However, the enhancement by the SMA is limited due to the weak bonding strength between SMA and the host. By acid and nano-silica particle treatment, the interfacial strength is increased compared with the initial one. The obtained results can be used as a guidance in the application of SMA-based smart structures.

## Figures and Tables

**Table 1:** Flexural modulus of SMA composite laminates with various ply modes (GPa).

-	No Fiber	2 Fibers	4 Fibers	6 Fibers	10 Fibers
Ply Mode I	22.73	23.81	23.91	22.42	22.11
Ply Mode II	22.73	23.61	23.73	22.38	21.66
Ply Mode III	22.73	22.74	23.74	22.21	-

**Table 2:** Variation of the interfacial strength of SMA/epoxy composites after being modified by 2 wt % nano-silica and different acid immersion durations in the pull out test.

**Immersion Duration/h**	0	2	4	6	8	10
**Shear Strength/MPa**	1.32	1.53	1.56	1.84	1.89	1.36
